# MDMA-assisted psychotherapy for PTSD in adolescents: rationale, potential, risks, and considerations

**DOI:** 10.1007/s00787-023-02310-9

**Published:** 2023-10-10

**Authors:** Samuli Kangaslampi, Josjan Zijlmans

**Affiliations:** 1 Faculty of Social Sciences/Psychology, Tampere University, Tampere, Finland; 2 Department of Neuropsychology and Psychopharmacology, Faculty of Psychology and Neuroscience, Maastricht University, Maastricht, The Netherlands; 3 Department of Child and Adolescent Psychiatry & Psychosocial Care, Amsterdam University Medical Center, Location Vrije Universiteit Amsterdam, Amsterdam, The Netherlands; 4 Amsterdam Public Health, Amsterdam University Medical Center, Mental Health, Amsterdam, The Netherlands

**Keywords:** MDMA, Psychotherapy, Adolescents, PTSD

## Abstract

3,4-Methylenedioxymetamphetamine(MDMA)-assisted psychotherapy (MDMA-AP) is a proposed treatment for posttraumatic stress disorder (PTSD) that may be approved for adults soon. PTSD is also common among trauma-exposed adolescents, and current treatments leave much room for improvement. We present a rationale for considering MDMA-AP for treating PTSD among adolescents. Evidence suggests that as an adjunct to therapy, MDMA may reduce avoidance and enable trauma processing, strengthen therapeutic alliance, enhance extinction learning and trauma-related reappraisal, and hold potential beyond PTSD symptoms. Drawing on existing trauma-focused treatments, we suggest possible adaptations to MDMA-AP for use with adolescents, focusing on (1) reinforcing motivation, (2) the development of a strong therapeutic alliance, (3) additional emotion and behavior management techniques, (4) more directive exposure-based methods during MDMA sessions, (5) more support for concomitant challenges and integrating treatment benefits, and (6) involving family in treatment. We then discuss potential risks particular to adolescents, including physical and psychological side effects, toxicity, misuse potential, and ethical issues. We argue that MDMA-AP holds potential for adolescents suffering from PTSD. Instead of off-label use or extrapolating from adult studies, clinical trials should be carried out to determine whether MDMA-AP is safe and effective for PTSD among adolescents.

3,4-Methylenedioxymetamphetamine-assisted psychotherapy (MDMA-AP) is a combination of psychopharmacology and psychological intervention used to treat posttraumatic stress disorder (PTSD). Phase 3 clinical trials of MDMA-AP for the treatment of PTSD among adults have finished, and U.S. Food and Drug Administration approval looks likely in the coming year [1, 2]. The potential, limitations, risks, and best practices of this form of treatment for adults are under active discussion [3–8]. However, adolescents also suffer from high rates of PTSD and while evidence-based psychological treatments exist, there is still a large group of young patients for whom treatment is ineffective. To alleviate unnecessary suffering and prolonged symptoms, new forms of treatment are called for.

If MDMA-AP is approved as a treatment for PTSD, the question of extending it to adolescents will soon follow and it is prudent to discuss and study the potential risks and benefits of extending treatment to adolescents beforehand. Here, we present a rationale for considering MDMA-AP for adolescents, provide initial observations and ideas about what researchers and clinicians might need to pay special attention to if MDMA-AP were to be used with adolescents, and point out areas where we especially need more research and information.

We will limit our discussion to adolescents of approximately 14–17 years of age. We will further only consider MDMA-AP for PTSD specifically (for some of the related issues in possibly extending psychedelic-assisted treatment to minors, see [9, 10]).

## Rationale and potential benefits

Posttraumatic stress disorder (PTSD) is unfortunately not uncommon among adolescents. More than half of adolescents may face a potentially traumatic event before they turn eighteen [11, 12]. Adolescents are generally resilient in the face of trauma. Still, depending on a host of peri-traumatic and posttraumatic factors, between 5 and 25% of those exposed go on to develop long-lasting, clinically relevant symptoms of PTSD [12–15].

Several evidence-based psychological treatments for PTSD among adolescents have been developed. The best evidence exists for the efficacy of trauma-focused cognitive-behavioral treatments [16–18], such as Trauma-Focused Cognitive Behavioral Therapy (TF-CBT [19]), Prolonged Exposure for Adolescents (PE-A [20]), Narrative Exposure Therapy (NET/KIDNET [21, 22]), and (developmentally adapted) Cognitive Processing Therapy (CPT/D-CPT [23, 24]). These treatments are effective, resulting in large improvements in PTSD symptoms on average [17, 25]. However, even in carefully conducted clinical trials around 11% of minor participants drop-out during treatment [26], with adolescents even more likely to drop-out than children [27]. Avoidance, number of traumatic events experienced, and quality of adolescent-therapist relationship further appear to predict drop-out [27–29]. In addition, between a quarter to half of participants fail to respond adequately to treatment [27, 30–32]. Higher age and high levels of PTSD symptoms predict non-response [27], but more specific reasons may include inadequate emotional engagement and non-constructive forms of processing the traumatic experiences during treatment [18, 19, 33]. Much could still be achieved by making existing treatments more widely available for adolescents suffering from PTSD. However, alternative treatment options are also needed, especially for adolescents who are not able to complete current treatments, or do not benefit adequately from them.

Trials of MDMA-AP for PTSD have found the treatment effective in reducing PTSD symptoms and promoting remission in adults [2, 34]. Still, it is not yet clear how MDMA-AP would fare in a direct comparison to the trauma-focused psychological treatments treatment guidelines currently recommend for PTSD, such as PE, CPT, or NET. Meta-analyses on these trauma-focused treatments have found similar-sized effects on symptoms and remission in adults as those reported in the most recent Phase 3 trial of MDMA-AP [5], although high drop-out rates of up to 30–50% are also reported [35, 36]. Effect sizes are roughly similar for trauma-focused treatments among adolescents [16, 18], though we do not yet know how MDMA-AP for adolescents would fare. Nonetheless, there are reasons to suspect that MDMA-AP could be more effective or better tolerated as a treatment option, or be effective for a group of adolescents that now fail to respond to therapy.

First, MDMA may reduce avoidance and enable trauma processing for individuals who feel unable to revisit their traumatic memories or are too overwhelmed by negative emotions to engage with them when they try to do so. By reducing anxiety and stress reactivity [37, 38], as well as detection of and responsivity to negative emotional information [37, 39, 40], while enhancing mood and receptiveness [37, 41, 42], MDMA could both attenuate overly negative reactions to traumatic memories [43] and help people tolerate such reactions. In addition, increased overall arousal [37, 44] and comfort when discussing emotional memories [45] under MDMA may increase motivation to engage, while reducing the risks of emotional numbing, overmodulation, avoidance, and dissociation that would prevent adequate emotional engagement.

These effects may hold special importance for traumatized adolescents, for whom difficulties in treatment adherence, dropouts, and motivation to engage and remain in treatment are pronounced [20, 23, 46]. In particular, the attenuation of overwhelming emotional reactions that could lead to impulsive withdrawal from treatment or harmful avoidance/coping behaviors could be of great benefit in treating traumatized adolescents who often have challenges in emotion regulation [47]. Furthermore, as elaboration, involvement in therapy, and maintaining an appropriate level of emotional engagement appear key to processing of traumatic experiences and symptom reduction in trauma-focused treatment of adolescents [20, 33, 48], effects of MDMA that promote disclosure and engagement and reduce avoidance could enhance treatment efficacy.

Second, overly negative, maladaptive beliefs and appraisals of the traumatic event and its sequelae are closely associated with PTSD symptoms among adolescents [49, 50] and improvement in such cognitions appears to be a mechanism of change involved in many forms of treatment for PTSD among both adults and adolescents [51, 52]. Reappraising the traumatic event, its characteristics, and consequences both under the acute influence of MDMA and in integrative sessions could aid in disconfirming and changing these problematic cognitions. In particular, feelings of safety and ability to confront traumatic material during MDMA sessions [53] may disconfirm beliefs of the world as a wholly dangerous place and the self as incompetent and weak, common to traumatized children and adolescents [54]. As MDMA promotes empathy and prosociality [41, 55, 56], it may reduce effects of perceived social rejection [57], and appears to sometimes promote insights and radical changes in perspectives [53, 58]. Processing trauma during MDMA sessions could thus also affect particularly challenging posttraumatic emotions like shame and guilt.

Third, MDMA appears to enhance fear extinction and/or disrupt fear memory reconsolidation [59–63], processes central to treatment of PTSD by psychological interventions [51, 64, 65]. In particular, MDMA may foster better inhibitory learning [66, 67] and affect reconsolidation because of the greater expectancy violation (or prediction error) induced by its mood-enhancing, fear attenuating, and prosocial effects [42, 68] as well as effects on recollection of negative memories [63, 69]. More effective fear extinction could mean better and faster relief from symptoms when adolescent patients confront their traumatic memories under MDMA. Shorter overall treatment duration or more immediately obvious improvement in symptoms would be important for adolescents whose patience and motivation for treatment is likely to fluctuate.

Fourth, therapeutic alliance has been found a key factor in the success of trauma-focused treatment of adolescents [70] and in reducing dropouts [28, 71]. Establishing a trusting therapeutic relationship may be especially challenging when working with adolescent patients who have experienced interpersonal trauma and the associated deep betrayal of trust [72]. MDMA may aid in strengthening the therapeutic alliance by promoting increased emotional empathy, trust, and closeness [42, 43, 55, 56]. The therapeutic relationship potentially strengthened by the effects of MDMA could also act as a corrective experience for negative beliefs about other people and their trustworthiness. Direct evidence of the post-acute effects of MDMA on therapeutic alliance is limited to clinical observations, however (e.g., [73, 74]).

Fifth, effects of MDMA-AP that go beyond PTSD symptoms may also hold relevance for potential use in adolescents. Though trials of MDMA-AP focus on PTSD symptoms, they have also found anti-depressive effects [2, 75], significant for adolescents among whom PTSD has very high comorbidity with depression [76]. To the extent that MDMA-AP promotes change in trauma-related cognitive processes such as maladaptive appraisals and trauma memory qualities through effects described above, its therapeutic effects for adolescent posttraumatic psychopathology could extend beyond PTSD symptoms [54, 77]. Trials of MDMA-AP for PTSD in adults have not included measures of changes in these processes so far, so comparisons with existing treatments are not possible. Adult trials do, however, suggest that MDMA-AP treatment may also help with problematic alcohol use [78] and improve sleep quality [79], both of which are significant issues in relation to PTSD in adolescents [80, 81].

In sum, we have identified five potential benefits of MDMA-AP for the treatment of adolescents with PTSD: MDMA-AP may (1) reduce avoidance and enable trauma processing, (2) aid in the reappraisal of beliefs, (3) enhance fear extinction, (4) strengthen therapeutic alliance, and (5) have beneficial effects beyond relief of PTSD symptoms. These potential benefits are generic to MDMA-AP, but some may be particularly useful in the treatment of adolescents. For example, as therapeutic alliance is important to successful trauma therapy and is hard to establish with adolescents, a form of treatment that would enhance this process would potentially be more effective in adolescents than in adults. In addition, treatment at a younger age may reduce lifetime disease burden and costs to adolescents and society, while saving quality-adjusted life years [82, 83].

## Considerations and adaptations

In MDMA-AP, MDMA itself is typically framed as a “therapeutic adjunct” or “catalyst” to a psychotherapeutic process [84, 85]. Thus, despite involving psychopharmacology, MDMA-AP would be a psychological intervention in the first place. Recently, arguments have been made that the psychotherapy component of MDMA-AP has received inadequate attention [6]

In most trials so far, the psychotherapy or psychological support component of MDMA-AP has followed the largely non-directive and flexible approach described in *A Manual for MDMA-Assisted Psychotherapy in the Treatment of Posttraumatic Disorder* [85], written mainly by Michael C. Mithoefer and published by the Multidisciplinary Association for Psychedelic Studies. This approach and manual were developed primarily based on early work with psychedelics and MDMA, especially that of LSD psychotherapy pioneer Stanislav Grof [86], rather than on theories or treatment modalities specific to PTSD [85]. Accordingly, although it acknowledges similarities with approaches like PE and cognitive restructuring, it also includes elements like focused bodywork and therapeutic use of music that are not found in other PTSD treatments.

Overall, rather than focusing on specific techniques and treatment elements, the manual strongly emphasizes a non-directive approach trusting the patient’s “inner healing intelligence” and “innate wisdom about their own healing process” [85]. It defines core elements of MDMA-AP but allows much leeway for “individual therapist teams to include therapeutic interventions based on their own training, experience, intuition, and clinical judgment” [85]. Accordingly, the manual suggests that “the successful use of MDMA in therapy depends on the ‘sensitivity and talent of the therapist who employs [it]’ (Grinspoon and Doblin, 2001)” [85]. This approach may have its benefits, for example in terms of flexibility in responding to patient needs. However, for TF-CBT, a clearly structured, defined, and manualized therapy, studies have found that therapist characteristics such as theoretical orientation, professional background, and clinical experience do not appear to contribute to the efficacy of PTSD treatment in children and adolescents [87, 88]. A more defined and specified format might lead to more uniform efficacy, less dependent on therapist characteristics, for MDMA-AP among adolescents, too, although this remains a hypothesis to be tested at this stage.

Considering what we know about how adolescents heal from posttraumatic symptoms and how current psychological treatments work, the psychotherapeutic component of MDMA-AP can probably be refined. In the potential application of MDMA-AP for adolescents, researchers and clinicians can look to existing adolescent adaptations of trauma-focused treatments for inspiration in several areas.

First, existing treatments like developmentally adapted CPT and PE-A stress the importance of motivation building when treating PTSD among (older) adolescents [20, 23]. Compared with children, adolescents’ motivation to engage and remain in treatment depends more strongly on the adolescents themselves than on their parents, but compared with adults, their motivation is more likely to fluctuate. Strive for autonomy combined with PTSD-related avoidance can lead to ambivalence toward treatment for traumatized adolescents [46], contributing to drop-out and premature treatment cessation [26, 71]. A motivational interview, openly discussing the impact of the trauma and the advantages and disadvantages of treatment, as well as actively challenging problematic ideas about therapy may be valuable avenues to prevent dropouts and maintain motivation in tailoring MDMA-AP for adolescents [20, 46]. The importance of detailed, age-appropriate psychoeducation (of trauma and trauma symptoms, the treatment plan and its rationale, and even issues like rights and justice) for motivation and collaboration is also emphasized by all trauma-focused treatments (e.g. [20]).

Second and relatedly, the development of a strong therapeutic alliance and relying on youth, rather than therapist, evaluations of its quality appear especially important for successful PTSD treatment among adolescents [70, 71, 89], and is emphasized in several treatment modalities, especially TF-CBT [19]. Therapeutic alliance and trust are already set out as core elements in MDMA-AP [85], but even more time and effort should probably be focused on developing the therapeutic alliance when working with adolescents in general, and especially when working with adolescents exposed to early interpersonal trauma [72]. Based on research findings and concerns young people have about trauma treatment, this time should especially be spent on emphasizing and enhancing the collaborative nature of the work ahead, addressing trust and confidentiality issues, and building rapport, while carefully attending to the adolescent’s perspective and demonstrating empathy and warmth [90–93]. The groundwork for a successful alliance will undoubtedly be laid in the initial non-drug preparatory sessions, but as pointed out before, MDMA sessions could hypothetically further strengthen the therapeutic alliance throughout the therapy.

Third, traumatized youth are likely to experience difficulties in emotion regulation [47]. Combined with the intense emotional life and rapid mood changes of all adolescents, and elevated potential to react to intense emotions with self-harm or substance abuse, this may call for more focus on emotion and behavior management techniques during treatment. The only regulation technique presented in the current MDMA-AP manual is diaphragmatic breathing [85]. In developing MDMA-AP for adolescents, interventions and techniques for emotion and behavior regulation could be adapted from, e.g., dialectical behavior therapy for PTSD [94], as was done for D-CPT [23], or from the first phase of the TF-CBT protocol [19]. Such techniques would also be helpful for adolescents suffering from PTSD who experience significant dissociative symptoms [95]. If risks of symptom exacerbation or problematic coping methods during or after the coming treatment sessions appear elevated for a particular adolescent patient, a crisis coping plan could be prepared beforehand [20].

Fourth, adolescents tend to be more impulsive [96], less able to sustain attention [97], and more distractible [98], suggesting they may need additional support to maintain therapeutic focus and reduce avoidance. This might conflict with the non-directive approach prescribed in the MDMA-AP manual where listening and presence are the main activities of the therapist. Therapists are instructed to employ “invitation rather than direction” and “minimal encouragement” [85] during MDMA sessions, although guidance or redirection is suggested in some circumstances. In adolescents, for some part of the MDMA session(s), deliberate, systematic, and gently guided exploration of trauma-related memories, thoughts, and emotions might be preferable to trusting the “inner healing intelligence” [85] and the patient’s self-direction. Here, the emphasis in especially NET [22, 99] and PE-A [20] on coherent narration of the traumatic memory and integration of autobiographical, temporal, and spatial information into a consistent declarative representation of the event(s) may be a valuable consideration when developing and trialing MDMA-AP for adolescents. In both approaches to imaginal exposure, the therapist guides and encourages the participant to focus on all aspects of the traumatic memories, including the most avoided and troubling parts that may be characterized by lack of coherence or organization and dominated by sensory and emotional elements. Explicitly observing similarities in emotions, thoughts and physical reactions that arise during treatment with those at the time of trauma could be a further valuable exercise during MDMA sessions and promote insight [22].

Besides potentially better organizing the traumatic memories [20–22], comprehensively activating and exploring them in a safe and empathetic encounter (both elements potentially strengthened by MDMA) can lead to corrective experiences and learning that trauma-affected maladaptive perceptions and beliefs are inaccurate, overly negative and catastrophizing. This active exposure-based work requires patience and resolve from therapists to let and even encourage adolescents to explore their traumatic memories, including their worst moments—a process that is bound to involve difficult emotions and reactions, hopefully made more manageable with the help of MDMA. Due especially to enhanced fear extinction and disrupted fear memory reconsolidation, several researchers have suggested the MDMA-induced state would be well suited for exposure-based work [3, 8, 100], but direct evidence awaits future (comparative) trials.

Especially when working with adolescents with multiple traumatic experiences, adopting the sort of life story perspective emphasized by NET, where traumatic experiences are explicitly placed in their own place and time and positive memories and experiences are also discussed could be valuable for the therapeutic process overall, including both MDMA and integrative sessions [22]. For exploring positive memories as sources of resilience and meaning, the acute effects of MDMA may be mixed, as there is evidence of enhanced vividness and positive affect when thinking about positive autobiographical memories [43] but also of amnestic effects on detailed positive recollection [69].

Fifth, as part of the therapeutic process, adolescents might need more support in dealing with social and developmental challenges and barriers to treatment not directly related to their trauma, as well as in practical integration of treatment benefits and separation from the therapist. Here, elements such as systematic evaluation of adolescent (and family) difficulties besides PTSD, plans for coping with crises and relapses, and final projects could be adapted from the more extensive case management and relapse prevention components included in PE-A, for example [20].

Finally, laws and definitions vary, but in many countries, older adolescents can make decisions on their medical care and give informed consent for research themselves (they are considered “mature minors” [101]; see also [102] for criticism and [103] for overview), and even ask for their parents not be informed of their choices. However, this does not mean that involvement of parents or guardians in the decision-making and treatment process would be unwelcome. On the contrary, in many cases, the involvement of parents or guardians could make an important positive contribution, including less likelihood of drop-out [28]. Of existing treatments, TF-CBT dedicates up to half of the treatment sessions as parent or conjoint sessions and offers many ideas about parent involvement [19]. In PE-A, tailored family involvement is also emphasized [20], while in D-CPT, sessions with caregivers are optional [23]. At the very minimum in MDMA-AP for adolescents, ample psychoeducation should be directed at parents and families so that they may best support the adolescent during and after treatment. Conjoint (non-drug) sessions with parents especially in the early and closing stages of the treatment could be considered, or parents could join in the concluding parts of MDMA sessions when drug effects have waned, as the current manual suggests for significant others [85].

On the other hand, adolescence is also a time of struggling for independence and autonomy and even conflict with parents. Some older adolescents may not want to involve their parents deeply in the treatment or share their intimate feelings or experiences with them [23]. Their wishes should be respected, and confidentiality maintained, barring legal or ethical requirements to disclose information in special circumstances of course [46]. With adolescents, it is their own motivation to accept treatment and remain in it that is crucially important and needs to be supported [23].

In sum, the unique characteristics of adolescents call for a reconsideration of the psychotherapeutic component of MDMA-AP, perhaps even developing an entirely new protocol for it. Here, we have argued for focusing on (1) ways of reinforcing motivation, (2) the development of a strong therapeutic alliance, (3) additional emotion and behavior management techniques, (4) more directive, exposure-based methods during MDMA sessions, (5) more support for concomitant challenges and integrating treatment benefits, and 6) involving family in treatment. These suggested adaptations are, admittedly, based on reasoning from indirect evidence. Clinical trials with a comparative or dismantling focus are needed to determine whether they are, in fact, useful or necessary. Alternatively, we could study the potential of directly combining MDMA with existing, evidence-based treatments already adapted to adolescents, such as TF-CBT or PE-A, as others have suggested for adults [3, 8] and trialed with good results for cognitive-behavioral conjoint therapy [104].

## Potential risks particular to adolescents

The average age of participants in recent trials of MDMA-AP has been around forty [2, 34]. Thus, their findings regarding safety and possible risks cannot be directly applied to 14–17-year-olds, and potential risks particular to adolescents should be considered.

First, the acute physiological effects of MDMA at typical doses used in therapeutic research, 75–125 mg, are well established among adult healthy volunteers [105] and include elevated heart rate, blood pressure, and body temperature. However, to our knowledge, human research with MDMA has never been carried out in adolescents. In young adults of age 18–45 years, Studerus et al. [106] did find younger age to predict higher heart rate elevation, although the authors did not consider this of much clinical or practical relevance. At face value, there seems to be no specific reason to consider these typical physiological effects or common side effects such as muscle tightness, sweating, headaches, or nausea [2, 4] significantly more or less problematic for adolescents than for adults, provided adequate pre-screening for risk factors such as cardiovascular disease is in place. Still, “young people are often not the same as adults in terms of response to […] medications and their side effects.” [107]. Any clinical research on MDMA in adolescents should start from safety and tolerability. The lack of age-specific dosing information is a common unmet need in pediatric psychopharmacology [108], and differences in pharmacokinetics, for instance, may extend beyond the smaller size of adolescents.

Second, regarding unpleasant psychological effects, Studerus et al. [106] found high neuroticism and trait anxiety to predict feelings of dread and impaired control and cognition as reactions to MDMA among healthy adults. Anxiety seems to be the most common acute psychological adverse effect in MDMA-AP for PTSD [4]. Some participants also experience anxiety and low mood post-acutely and in the longer term. We agree with Breeksema et al. [4] on the importance of studying whether such effects are related to typical temporary emotional distress related to therapeutic processing of trauma or are better viewed as post-acute adverse effects due to MDMA. In any case, these findings could have relevance for adolescents, as neuroticism and negative emotionality is typically higher in adolescence before decreasing in young adulthood [109, 110]. Further, a few adverse events related to suicidality were reported in the latest Phase 3 trial of MDMA-AP among adults, in both the MDMA and placebo groups, although suicidal behavior only occurred in the placebo group [2]. Accordingly, the elevated incidence of self-harm among adolescents as well as suicidality in older adolescents, especially pronounced for those with histories of abuse or suffering from PTSD [111, 112] should also be considered. Although (trauma-focused) treatment of PTSD among adolescents has not been found to exacerbate suicidal ideation and may indeed reduce it [64, 113], close monitoring of adolescents throughout treatment and perhaps especially in the sub-acute phase is called for, with special attention to potential adverse events involving self-harm and suicidality.

Third, the potential toxicity of MDMA to the (developing) brain and possible cognitive harms are an important question for therapeutic use among adolescents. Heavy adult recreational MDMA users (typically with lifetime consumption in the hundreds of MDMA tablets) exhibit deficits in several areas of cognitive function, including attention and working and short-term memory, although it remains unclear whether these deficits are exclusively caused by MDMA use [114]. At high doses, MDMA is neurotoxic to animals, including non-human primates, leading to loss of neuronal bodies and terminals [115]. Some animal studies also suggest long-term cognitive deficits from high-dose MDMA exposure in adolescence [116, 117], although other studies did not find effects on cognition [114]. Findings on whether adolescent animals are less or more susceptible to MDMA-induced neurotoxicity than adults are mixed [118–122]. As most animal studies involve (repeated) high doses, their relevance for a few administrations of smaller, therapeutic doses is unclear. A systematic review of 90 animal experiments found no evidence that MDMA produces cognitive impairments in animals at doses below 3 mg/kg [114], whereas clinical dosages in humans are about 1 to 2 mg/kg. In their review, Pantoni and Anagnostaras [114] conclude that “overall, the preclinical evidence of MDMA-induced cognitive deficits is weak” and that “it is unlikely that less than 3 mg/kg of pure MDMA poses significant danger to neurological health if administered infrequently and in a controlled setting.” Still, as direct evidence in adolescents is lacking, it would be important to study potential cognitive harms in any clinical trial of MDMA-AP for adolescents.

Fourth, although dependence is very rare [123, 124], MDMA has some misuse potential among both adults and adolescents. Among adolescents with substance use disorders, there are further indications that those with PTSD are more likely to use MDMA illicitly, and that their MDMA use is associated with coping and self-medication motives, but also avoidance-type symptoms specifically [125, 126]. Trials of MDMA-AP with adults have not reported increased use of MDMA or indications of abuse potential among participants [2, 4]. However, increased risk-taking, reward sensitivity, and impulsivity in adolescence [96, 127, 128] may mean that the risk of misuse or wish to use MDMA beyond the clinical setting once aware of its effects could be elevated for adolescents, as compared with especially older adults [129].

Fifth, history presents us with examples of (by today’s standards) unethical psychopharmacological research on children, including with psychedelics [130] and stimulants [131]. In current MDMA-AP research with adults, at least one serious instance of unethical behavior has occurred [132]. Any clinical use or research involving MDMA in minors must adhere to high ethical standards and be cognizant of the complex ethical issues involved in child and adolescent psychopharmacology [103, 133], further complicated by the altered state of consciousness produced by MDMA. Here too, a more explicitly specified and structured format for the psychotherapeutic component of MDMA-AP could help in further defining expected and appropriate forms for adolescent-therapist interaction, especially during the dependent and vulnerable MDMA-induced state [4, 6].

Overall, a recent review suggested adverse events and harms may have been poorly defined and inadequately assessed in MDMA-AP studies [4]. Any studies among adolescents should pay close attention to extensively and openly reporting adverse events and harms. At the same time, any side effects or risks that do emerge must be considered in relation to how effective MDMA-AP turns out to be for addressing the enormous burden of untreated PTSD among adolescents.

## Conclusions

As many adolescents suffer from PTSD, and current evidence-based treatments leave room for improvement, there is a need for new approaches to address PTSD among adolescents. Considering evidence of efficacy among adults, and what we know about how MDMA might enhance treatment, we have argued here that it makes sense to study MDMA-AP for PTSD in adolescents. As a developmental period of great change and reorganization, adolescence represents both challenges and opportunities for mental health treatment. We have suggested potential modifications and adaptations to especially the psychotherapeutic component of MDMA-AP that could be useful for adolescents, and discussed potential risks and concerns specific to them, summarized in Table 1.

**Table 1:** Overview of potential benefits, considerations, and risks and unknowns of MDMA-assisted psychotherapy for adolescents with posttraumatic stress disorder

Benefits	Considerations	Risks and unknowns
Avoidance reduction; enabling of trauma processing	Treatment motivation	Age-specific dosing and physiological effects
Reappraisal of beliefs	Importance of therapeutic alliance	Adverse effects, including anxiety and self-harm
Enhanced fear extinction	Additional emotion and behavior management techniques	Misuse potential
Strengthened therapeutic alliance	More directive, exposure-based methods	Vulnerable position during treatment
Effects beyond PTSD symptom relief	More support for concomitant challenges and integrating treatment benefits	
	Family involvement	

If MDMA-assisted psychotherapy becomes an approved treatment for adults, it may be possible in many countries to extend use to adolescents, even if no studies among them have been carried out. For years, experts on pediatric psychopharmacology have emphasized and criticized the “frequent off-label prescription of medications to children and adolescents based exclusively on data from [RCT’s] involving adult patients” [108]. As Tan and Koelch [134] noted, due to the prominent ethical concerns and rigorous requirements for psychopharmacological research among minors, they are, paradoxically, sometimes so well protected against research that there is little data available about the safety and effectiveness of psychiatric medications they are commonly described. Accordingly, we should try to avoid a situation where MDMA-AP is applied to adolescents in an off-label and ad hoc basis, with the assumption that results among adults also apply to them.

Especially when psychopharmacology is involved, “for youth populations it is not sufficient to extrapolate efficacy or side effect data from adult studies” [107]. We should face the ethical and practical challenges and carry out the research to ensure that if MDMA-AP is used among adolescents, this practice is evidence-based. If MDMA-AP is approved for adults, trials with adolescents using the current treatment protocol are expected. We hope that these possible future trials will be complemented by comparative studies that vary and adapt the psychotherapeutic component, and that together they will either provide such an evidence base or show that MDMA-AP should not be used for adolescents in any form.

## Data Availability

Not applicable.
